# What are the major causes of lower limb amputations in a major Australian teaching hospital? The Queensland Diabetic Foot Innovation Project, 2006 – 2007

**DOI:** 10.1186/1757-1146-4-S1-O24

**Published:** 2011-05-20

**Authors:** Peter A Lazzarini, Damien Clark, Patrick H Derhy

**Affiliations:** 1 Allied Health Research Collaborative, Metro North Health Service District, Queensland Health, Brisbane, Australia; 2 Department of Podiatry, Metro North Health Service District, Queensland Health, Brisbane, Australia; 3 School of Public Health, Queensland University of Technology, Brisbane, Australia; 4 Department of Podiatry, Metro South Health Service District, Queensland Health, Brisbane, Australia; 5 Centre for Healthcare Improvement, Queensland Health, Brisbane, Australia

## Background

Lower extremity amputation (LEA) results in significant hospitalisation, rehabilitation, morbidity and mortality. In 2004, 3,400 LEAs, with an average length of stay of 26 days, were performed in Australia for diabetic foot complications alone. However, diabetic foot complications are commonly recognised as the most common cause of “non-traumatic” LEA internationally. Unfortunately, there seems to be a paucity of Australian data on total numbers and causes of all LEAs. This retrospective audit aimed to evaluate underlying primary indications for LEA at a major Australian tertiary hospital.

## Methods

Cases of LEA performed at the Princess Alexandra Hospital (Brisbane, Australia) between July 2006 and June 2007 were identified through the relevant hospital discharge dataset codes (n = 197). In all cases clinical records were audited for indications of diabetes, trauma and other major causes for LEA. Exclusion criteria included records unable to be accessed during the operation of the clinical audit and cases that were found to have multiple possible major indicators of LEA.

## Results

Eleven cases were excluded (9 inaccessible records and 2 multiple major indications). Cases included (n = 186) accounted for 160 unique patients. Overall mean age at LEA procedure was 67 years, 56% were first amputations, 54% were major amputations, and 9% died post-LEA in the audit year. Cases with diabetes indicated accounted for 60% of all LEAs, had a mean age at 70 years, 45% were first amputations, 50% were major amputations, and 7% died post-LEA. Primary indications for LEAs (n = 186) included: type 2 diabetes (53.2%), non-diabetes peripheral vascular disease (18.3%), trauma or accident (8.1%), type 1 diabetes (7.0%), cancer or tumour (5.4%), orthopaedic deformity (3.8%), and post-surgical emboli (2.2%).

## Conclusions

Most literature indicates that diabetes is the largest cause of “non-traumatic” LEAs. Our findings seemed to indicate that diabetes was the largest cause or indication of total LEAs. More statistical analysis between non-diabetes and diabetes LEA populations in regards to differences in age at LEA, major amputations, first amputations and post-LEA death will be presented.

